# An Update on Oligosaccharides and Their Esters from Traditional Chinese Medicines: Chemical Structures and Biological Activities

**DOI:** 10.1155/2015/512675

**Published:** 2015-03-15

**Authors:** Xiang-Yang Chen, Ru-Feng Wang, Bin Liu

**Affiliations:** School of Chinese Medicine, Beijing University of Chinese Medicine, Beijing 100102, China

## Abstract

A great number of naturally occurring oligosaccharides and oligosaccharide esters have been isolated from traditional Chinese medicinal plants, which are used widely in Asia and show prominent curative effects in the prevention and treatment of kinds of diseases. Numerous *in vitro* and *in vivo* experiments have revealed that oligosaccharides and their esters exhibited various activities, including antioxidant, antidepressant, cytotoxic, antineoplastic, anti-inflammatory, neuroprotective, cerebral protective, antidiabetic, plant growth-regulatory, and immunopotentiating activities. This review summarizes the investigations on the distribution, chemical structures, and bioactivities of natural oligosaccharides and their esters from traditional Chinese medicines between 2003 and 2013.

## 1. Introduction

Oligosaccharides and their esters, a significant group of phytochemical compounds, are widely distributed in the roots, rhizomes, stems, barks, leaves, aerial, and whole parts of medicinal plants. They not only serve as the energy storage components, but also play a vital role in the treatment of diseases. Before 2003, there have been a number of reviews and reports in respect to the isolation and structure elucidation of oligosaccharides and their esters from Chinese medicinal plants [1–3], but few biological activities such as cancer chemopreventive, and protein kinase C inhibitory activities had been reported [4–6]. With the development of isolation and identification techniques [7–11], a larger number of oligosaccharides and their esters have been endlessly identified from traditional Chinese medicines in the past decades. These compounds have a wide variety of structure types because of the assembly of different monosaccharide units, the combination of various linking styles and the existence of kinds of substituents. And more promising biological activities associated with some of the oligosaccharides and their esters have been discovered.* In vitro* and* in vivo* investigations have demonstrated that they displayed antioxidant, antidepressant, anti-inflammatory, neuroprotective, cerebral protective, antidiabetic, cytotoxic, antineoplastic, plant growth-regulatory, and immunopotentiating activities, and so forth. This review aims to provide a systemic summary of the studies on the distribution, chemical structures and biological activities of naturally occurring oligosaccharides and their esters from traditional Chinese medicines in the past decades. Among these compounds, the number of oligosaccharide esters is much greater than that of oligosaccharides, and the disaccharide esters are a very valuable source of active compounds. This information may help readers understand the structure characteristics and therapeutic indications of oligosaccharides and their esters from traditional Chinese medicines and offer clues to the development of new drugs.

## 2. Chemical Structures

Phytochemical investigations of traditional Chinese medicines have shown that many botanical families, including Polygalaceae, Liliaceae, Asteraceae, Polygonaceae, Smilacaceae, Scrophulariaceae, Asclepiadaceae, Arecaceae, Orobanchaceae, Acanthaceae, Rosaceae, Musaceae, Sparganiaceae, Leguminosae, Equisetaceae, Boraginaceae, Iridaceae, Alismataceae, Lamiaceae, Araliaceae, Rubiaceae, Oleaceae, Apocynaceae, Caryophyllaceae, Aspleniaceae and Trilliaceae, are rich in oligosaccharides and their esters. Oligosaccharides show diversified structures because of the type and the number of monosaccharides, as well as the position of glycosidic bonds. And oligosaccharide esters also display distinctive structural diversity largely owing to the number, type, and position of* O*-substituent units, including phenylpropanoid groups (e.g., coumaroyl, feruloyl, caffeoyl, sinapoyl, 3,4,5-trimethoxycinnamoyl, and cinnamoyl), benzoyl,* p*-methoxybenzoyl, and* p*-hydroxybenzoyl groups (Figure 2). Moreover, the double bonds of phenylpropanoid groups possess* trans* and* cis* isomeric forms, of which the* trans* forms widely exist in nature. Hence, according to the number of monosaccharides and the characteristics of chemical structures, these oligosaccharide esters could be categorized into 7 large groups.

### 2.1. Oligosaccharides

**Figure 1:**
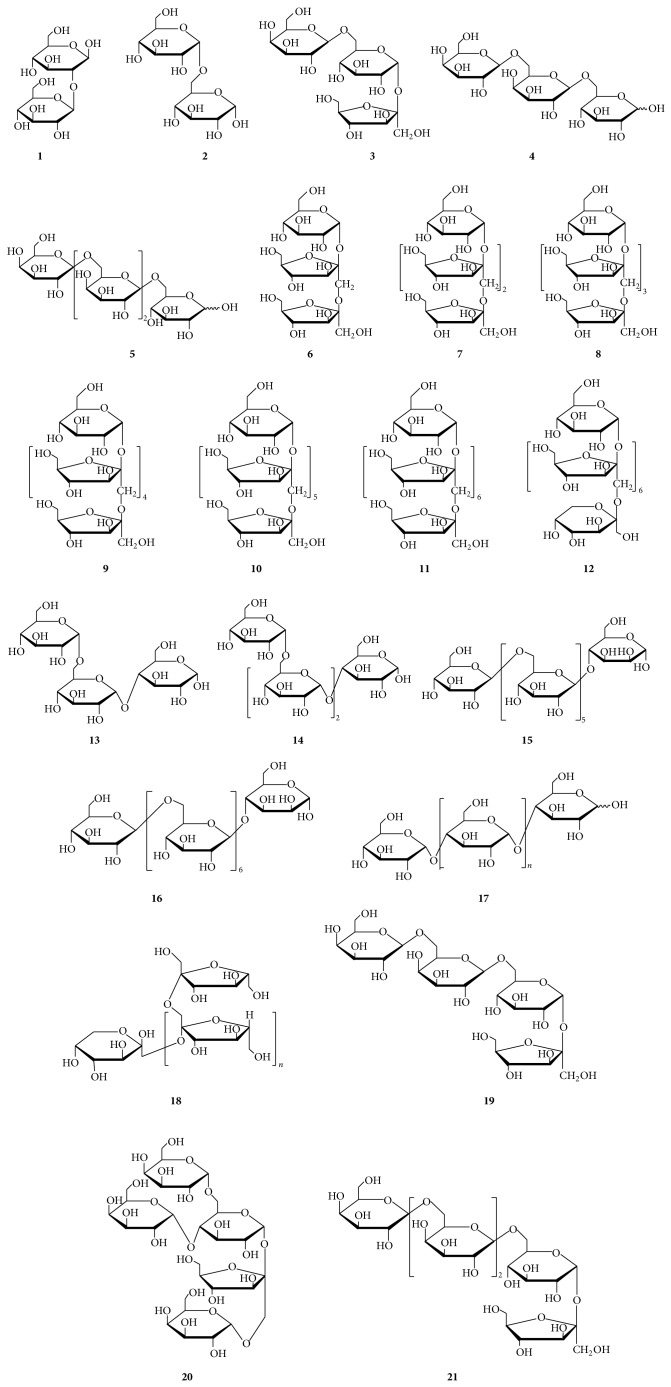


**Figure 2:**
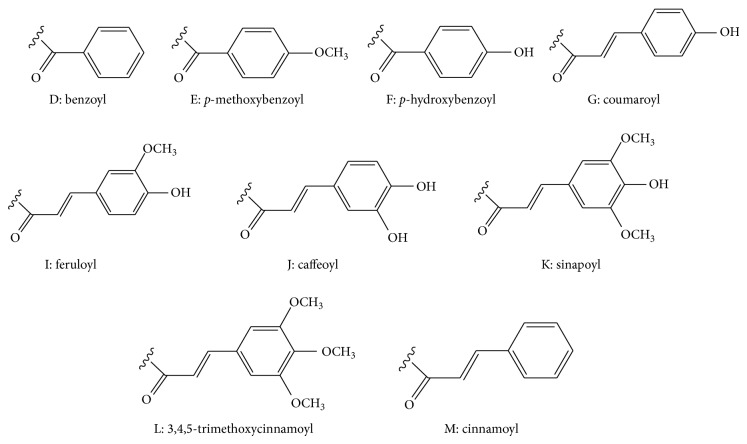


All compounds of this group (Table 1 and Figure 1) merely consist of various monosaccharides without* O*-substituents. In addition to the well-known sucrose, *β*-D-glucopyranosyl(1→2)-*β*-D-glucopyranoside (**1**) was isolated from* Camptosorus sibiricus *[12]. The oligosaccharides of raffinose (**3**), stachyose (**19**), and verbascose (**21**), all of which belong to the Raffinose family, possess one, two or three galactopyranosyl units linked to sucrose and have been found in the rhizomes and roots of* Alisma orientalis* [13],* Lycopus lucidus* [14],* Rehmannia glutinosa* [15, 16],* Salvia miltiorrhiza* [17], and* Scrophularia ningpoensis* [18]. Manninotriose (**4**) and verbascotetraose (**5**) consisting of galactopyranosyl units and a glucopyranosyl unit have been isolated from* Alisma orientalis* [13].

Five oligosaccharides comprising 1-kestose (**6**), nystose (**7**), 1-*β*-fructofuranosylnystose (**8**), hexasaccharide (**9**), and heptasaccharide (**10**) consisting of fructofuranose and glucopyranose have been isolated from the aerial parts and roots of* Gynura divaricata* subsp.* formosana* [19],* Morinda officinalis* [20–22],* Saussurea lappa* [23], and* Aralia cordata* [24]. Two water-soluble oligosaccharides (**11**,** 12**) composed of two or three types of monosaccharides including glucopyranose, fructopyranose, and fructofuranose have been obtained from the whole plants of* Blumea riparia* [25, 26].

Besides, malto-oligosaccharides (**17**, *n* = 0~8) consisting of *α*-D-glucopyranosyl residues assembled by (1→4)-linkages and inulo-oligosaccharides (**18**, *n* = 1~3) consisting of only fructosyl residues formed by (2→1)-linkages have been found in the roots of* Panax ginseng* [27, 28] and* Morinda officinalis* [20], respectively. Three noteworthy oligosaccharides (**2**,** 13**,** 14**) formed by *α*-D-glucopyranosyl units with (1→6)-linkages and a (1→4)-linkage have been found in the roots of* Panax ginseng* [28]. And two linear oligosaccharides termed heptasaccharide (**15**) and octasaccharide (**16**) consisting of glucose and mannose monomers were identified from the rhizomes of* Paris polyphylla* var.* yunnanensis* [29, 30]. A pentasaccharide, stellariose (**20**) consisting of a raffinose backbone with two galactosyl residues bound to the fructosyl and glucosyl moieties was identified from the stems of* Stellaria media* [31].

Oligosaccharides (Table 1) are composed of seven kinds of deoxyhexoses including cymaropyranose, canaropyranose, digitoxopyranose, oleandropyranose, digitalopyranose, cymaropyranurolactone, and oleandronic acid-*δ*-lactone. Oleandronic acid-*δ*-lactone exhibits the boat and chair conformations. The hydroxyl, methyl, and acetyl groups are located at the equatorial (e) and axial (a) bonds in the chair conformation of deoxyhexoses. These oligosaccharides were isolated from the traditional Chinese medicines including the roots of* Periploca forrestii*, the root barks of* P. sepium*, the stems of* P. calophylla,* and the barks of* Parabarium huaitingii*.

### 2.2. Oligosaccharide Esters

#### 2.2.1. Phenylpropanoid-Derived Disaccharide Esters

**Figure 3:**
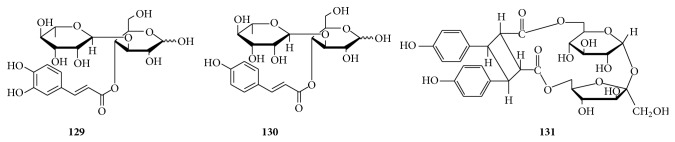


Phenylpropanoid-derived disaccharide esters (Table 2 and Figure 3) account for a considerable proportion of oligosaccharide esters and mainly possess a core of sucrose carrying a varying number of* O*-substituents, including phenylpropanoid groups, acetyl, benzoyl,* p*-methoxybenzoyl, and* p*-hydroxybenzoyl groups. Phenylpropanoid substituents are just present at 1′, 3′, 4′, 6′ positions of *β*-D-fructofuranosyl unit in compounds** 35**–**97**, whereas they appear at 2, 3, 4, 6 positions of *α*-D-glucopyranosyl moiety in compounds** 98**–**101**. Moreover, compounds** 76**–**97** are mainly esterified with acetyl groups along with a phenylpropanoid substituent, coumaroyl, feruloyl, or 3,4,5-trimethoxycinnamoyl group. Interestingly, the phenylpropanoid substituents are only attached to the 3′ position of sucrose. The two sugar rings of compounds** 102**–**128** both possess phenylpropanoid substituents. These oligosaccharide esters have been found in the roots and rhizomes of* Polygala tricornis*,* P. tenuifolia*,* Fagopyrum tataricum*,* Scrophularia ningpoensis*,* Cynanchum amplexicaule*,* Smilax riparia*,* Paris polyphylla *var.* yunnanensis*,* Smilacis glabrae*,* Fagopyrum dibotrys*, and* Sparganium stoloniferum*, the underground parts of* Trillium kamtschaticum*, the stems of* Polygonum sachalinensis*,* P. cuspidatum*,* P. hydropiper*,* Smilax china*, and* Calamus quiquesetinervius*, the aerial parts of* Polygala sibirica*,* Smilax bracteata*,* Heterosmilax erythrantha*, and* Musella lasiocarpa*, the leaves of* Persicaria hydropiper* and* Polygonum hydropiper*, the whole plants of* Bidens parviflora* and* Polygala hongkongensis*, and the flower buds of* Prunus mume*.

Cistanoside F (**129**) has been found in the stems of* Cistanche tubulosa* [80] and* C. sinensis* [81], the barks of* Paulownia tomentosa *var.* tomentosa *[82], and the aerial parts of* Acanthus ilicifolius* [83]. Cistanoside I (**130**) has also been isolated from the stems of the* Cistanche* plants [84]. Both of them are composed of glucosyl and rhamnosyl groups connected by a 1→3 glycosidic bond. In addition, 6,6′-sucrose ester of (1*α*,2*α*,3*β*,4*β*)-3,4-bis(4-hydroxyphenyl)-1,2-cyclobutanedicarboxylic acid (**131**) with a bis(4-hydroxyphenyl) cyclobutanedicarboxyl group as the acyl unit in the molecule structure was isolated from the whole plants of* Bidens parviflora *[61].

#### 2.2.2. Fatty Acid-Derived Disaccharide Esters

Eight monosubstituted disaccharide esters (Table 3) with a sucrose moiety possess six different types of fatty acid residues which attach to the 6 or 6′ position of sucrose. These fatty acids include linoleic acid, palmitic acid, linolenic acid, myristic acid, hexadeca-7,10,13-trienoic acid, and hexadeca-7,10-dienoic acid. The above sucrose fatty acid esters have been found in the rhizomes of* Astragalus membranaceus* and the roots of* Equisetum hiemale*.

#### 2.2.3. Lignan-Derived Disaccharide Esters

**Figure 4:**
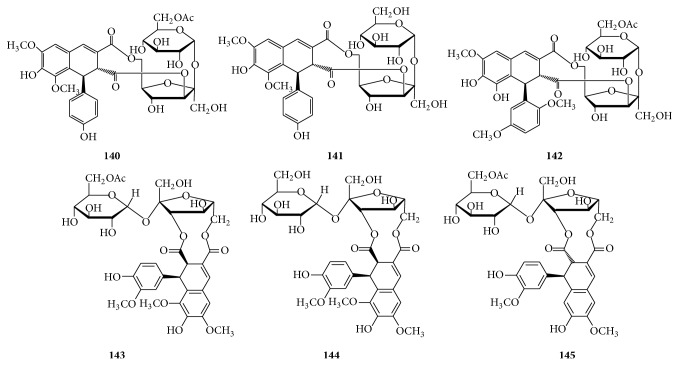


Six lignan-derived disaccharide esters (Figure 4) contain a sucrose core esterified with different lignan residues covalently linked to the 3′ and 6′ positions of *β*-D-fructofuranosyl unit and have been isolated from the whole parts of* Trigonotis peduncularis* [85] and the aerial parts of* Eritrichium rupestre* [86].

#### 2.2.4. Phenylpropanoid-Derived Trisaccharide Esters

**Figure 5:**
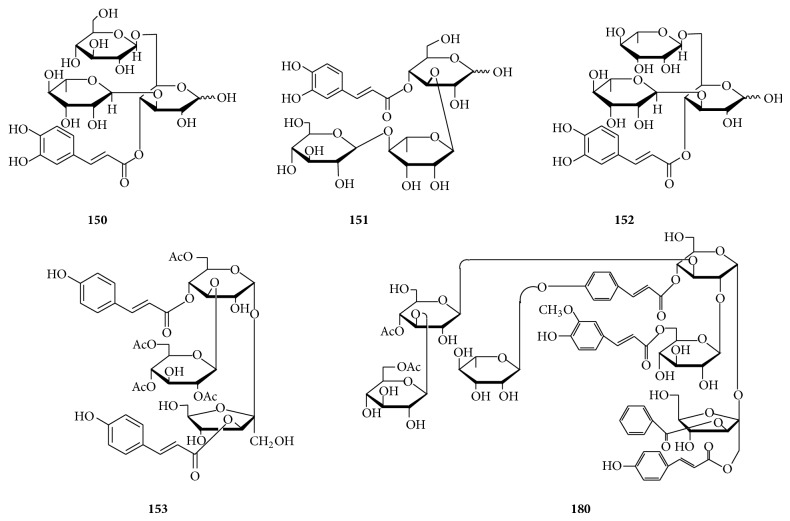


In this group, all oligosaccharide esters consist of three monosaccharides including glucopyranose, fructofuranose, and rhamnopyranose with* O*-substituents, which comprise feruloyl, sinapoyl, and 3,4,5-trimethoxycinnamoyl groups. Oligosaccharide esters (**146**–**149**) listed in Table 4 as well as kankanose (**150**), cistantubulose A_1_/A_2_ (**151**), cistansinensose A_1_/A_2_ (**152**), and compound** 153** shown in Figure 5 have been found in the roots of* Polygala tricornis* [37], the stems of* Cistanche tubulosa* [81, 87] and* C. sinensis* [80], the whole parts of* Boschniakia rossica* [88], and the rhizomes of* Iris brevicaulis* [89].

#### 2.2.5. Phenylpropanoid-Derived Tetrasaccharide Esters

Phenylpropanoid-derived tetrasaccharide esters (**154**–**159**) (Table 5) consisting of three glucopyranosyl units and a fructofuranosyl unit have been identified from the roots of* Polygala tricornis*. The nonsugar moieties of these oligosaccharides include coumaroyl, feruloyl, sinapoyl, and 3,4,5-trimethoxycinnamoyl groups.

#### 2.2.6. Phenylpropanoid-Derived Pentasaccharide Esters

As shown in Table 6, oligosaccharide esters (**160**-**179**) possessing a skeleton of five sugar residues have been isolated from the roots of* Polygala tenuifolia*. The sugar residues are composed of two types of monosaccharides including fructofuranose and glucopyranose, which are esterified with acetyl, benzoyl, rhamnose-substituted/nonsubstituted coumaroyl, and rhamnose-substituted/nonsubstituted feruloyl groups. Other than that, a structure-complex oligosaccharide polyester shown in Figure 5, polygalajaponicose I (**180**), consisting of a pentasaccharide backbone esterified with feruloyl, coumaroyl, rhamnosyl-coumaroyl, acetyl, and benzoyl groups has been obtained from the roots of* P. japonica* [90].

#### 2.2.7. Others

**Figure 6:**
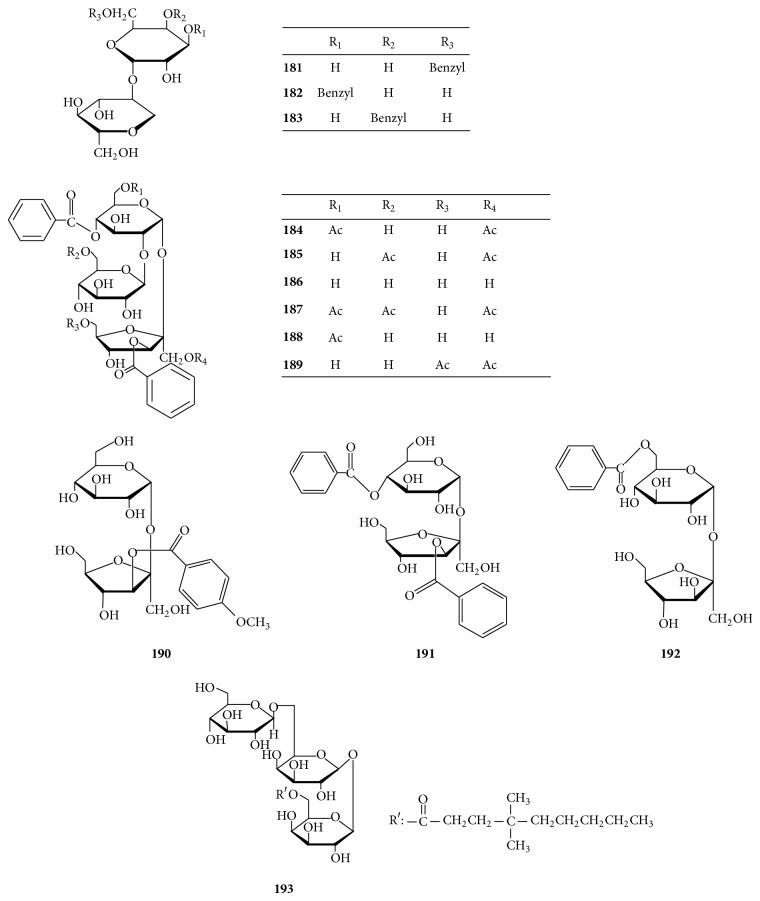


Polygalatenosides A–C (**181**–**183**) (Figure 6) containing a galactosyl unit and a polygolitosyl unit esterified with benzoyl groups at 3, 4 and 6 positions have been found in the roots of* Polygala tenuifolia* [91]. Three sucrose esters, including polygalatenoside D (**190**), telephiose F (**191**), and 6-*O*-benzoylsucrose (**192**), possess one benzoyl group, two benzoyl groups, and a* p*-methoxybenzoyl group, respectively. They were isolated from the roots of* P. tenuifolia* [91], the whole plants of* P. telephioides* [92], and the roots of* P. tricornis* [37]. Six trisaccharide esters, named telephioses A–E and G (**184**–**189**) with substituents of acetyl and benzoyl groups, were isolated from the whole plants of* P. telephioides* [92, 93]. Moreover, a trisaccharide ester (**193**), pubescenside A from the flowers of* Syringa pubescens*, possesses a fatty acid residue [94].

## 3. Biological Activities of Oligosaccharides and Their Esters

The oligosaccharides and oligosaccharide esters from Chinese medicinal plants are important products with diversified structures, which have triggered an increasing number of studies carried out on the isolated compounds. And thus diverse pharmacological activities have been proved. Among the isolated compounds, oligosaccharides, phenylpropanoid-derived disaccharide esters and trisaccharide esters, fatty acid-derived disaccharide esters, and others from the families Polygonaceae, Asclepiadaceae, Rubiaceae, Polygalaceae, Liliaceae, Smilacaceae, Arecaceae, Orobanchaceae, Scrophulariaceae, Acanthaceae, Rosaceae, Sparganiaceae, Leguminosae, and Equisetaceae have shown significant pharmacological activities including antioxidant, antidepressant, cytotoxic, antineoplastic, anti-inflammatory, antidiabetic, plant growth-regulatory, neuroprotective, and cerebral protective activities. Lignan-derived disaccharide esters, phenylpropanoid-derived tetrasaccharide esters, and pentasaccharide esters with biological activities have not been reported. Aside from the isolated constituents, oligosaccharide mixtures from* Rehmannia glutinosa*,* Panax ginseng*, and* Scrophularia ningpoensis *were also reported to display diverse pharmacological activities, such as antidiabetic, immunopotentiating, enhanced memory, and antineoplastic activities. These active compounds and mixtures could serve as the valuable candidates to be developed as possible drugs for the treatment and prevention of diseases.

### 3.1. Antioxidant Activity

The adverse effects of oxidative stress proposed to play significant roles in the pathogenesis of cardiovascular diseases, atherosclerosis, hypertension, cancer, diabetes mellitus, neurodegenerative diseases, rheumatoid arthritis, ischemia/reperfusion injury, and ageing have become an inevitable and serious issue [95, 96]. Scientists have thus made great efforts to explore antioxidants from medicinal plants by using different kinds of assay methods, which include DPPH radical scavenging assay, hydroxyl radical scavenging assay, superoxide anion scavenging assay, and ABTS radical scavenging method [96].

Lapathosides C and D, hydropiperoside, vanicoside B, hidropiperosides A and B, lapathoside A, and diboside A were isolated from the* Polygonum*,* Persicaria*, and* Fagopyrum* genera belonging to the Polygonaceae family. The DPPH test revealed that free radical-scavenging activity of the isolated compounds termed lapathoside C (**115**), hydropiperoside (**42**), vanicoside B (**111**), and lapathoside D (**38**) increased in turn, and lapathoside D exhibited strongest scavenging ability with an IC_50_ of 0.088 *μ*M [43]. Hidropiperosides A and B (**117**,** 118**) were reported to show obvious antioxidant response to DPPH radicals with the SC_50_ values of 23.4 and 26.7 *μ*g/mL, respectively, while vanicoside E moderately exhibited the same activity with a SC_50_ value of 49.0 *μ*g/mL [72]. Lapathoside A (**114**) and diboside A (**116**) just showed lower antioxidant activities with the SC_50_ values of 199.48 and 165.52 *μ*M, respectively [73].

Smiglasides A and B, smilaside P, 2,6-di-acetyl-3′,6′-di-feruloylsucrose, helonioside B, smilasides G–L, and heterosmilaside were isolated from the* Heterosmilax* and* Smilax* genera. Compared with ascorbic acid (IC_50_ 143.52 *μ*M) used as positive control, smiglasides A and B, and smilaside P (**48**,** 49**,** 65**) (IC_50_ 339.58, 330.66 and 314.49 *μ*M, resp.) showed higher antioxidant activities than 2,6-di-acetyl-3′,6′-di-feruloylsucrose (**73**) and helonioside B (**40**) (IC_50_ 631.66 and 518.27 *μ*M, resp.) [47]. Additionally, Nhiem et al. reported that helonioside B, heterosmilaside (**104**), and 2,6-di-acetyl-3′,6′-di-feruloylsucrose exhibited important DPPH radical scavenging activities with the SC_50_ values of 9.1, 12.7 and 8.7 *μ*g/mL, respectively [48]. Compared with smilasides G-I (**57**–**59**) (ED_50_ 68.5–79.4 *μ*M), smilasides J–L (**60**–**62**) showed higher radical scavenging activities with an ED_50_ value of 26.7–32.7 *μ*M [44].

Five quiquesetinerviusides A–E (**105**–**109**) isolated from the* Calamus* genus showed weak DPPH scavenging activities (IC_50_ 60.4–101.8 *μ*M) but exhibited better hydroxyl radical scavenging activities (IC_50_ 3.6–8.4 *μ*M). Moreover, quiquesetinerviuside C showed superoxide anion scavenging activity with an IC_50_ value of about 184.3 *μ*M [70]. Liu et al. investigated the antioxidant capacity of 3′,6-*O*-di-sinapoylsucrose (DISS) (**123**) by using the accelerated senescence-prone, short-lived mice (SAMP)* in vivo*. The analyses indicated that the activities of antioxidant enzymes of SOD and glutathione peroxidase ascended obviously in SAMP mice when amended with DISS 50 mg/kg. Moreover, DISS could downregulate and even restore the level of malondialdehyde in SAMP model group [97].

From the above studies, it can be concluded that oligosaccharide esters with antioxidant activities have been identified in the Polygonaceae, Liliaceae, Smilacaceae, and Arecaceae families. The results of the antioxidant assays show that the increased number of phenolic hydroxyl groups and acetyl groups could produce higher antioxidant activity. Fan et al. indicated that the increased number of phenylpropanoid groups was not beneficial to free radical scavenging activity [43]. Zhang et al. pointed out that oligosaccharide esters with feruloyl groups exhibited better antioxidant activities than those with coumaroyl groups [44].

### 3.2. Antidepressant Activity

The oligosaccharides obtained from the* Morinda* genus not only show specific antidepressant and antistress activities but also have no suppression or excitatory effects on central nervous system as well. What is more, they can be taken orally with little toxicity [21]. The inulin-type hexasaccharide (IHS) (**9**) from* Morinda officinalis* obviously exhibited cytoprotective activity, which contributed to the antidepressant effect, not only by providing the PC12 with protection against Cort-induced lesion with IHS 0.625 and 1.25 *μ*M, but also by reducing the Cort-induced [Ca^2+^]_*i*_ overloading with IHS 2.5 and 10 *μ*M. IHS 5 and 10 *μ*M upregulated the nerve growth factor mRNA expression in Cort-induced PC12 cells [22]. Polygalatenosides A (**181**) and B (**182**) were isolated from the* Polygala* genus. They significantly inhibited the isotope-labeled RTI-55 binding to norepinephrine transporter protein with the IC_50_ values of 30.0 and 6.04 *μ*M, respectively [91].

DISS and tenuifoliside A were isolated from the* Polygala* and* Cynanchum* genera. Liu et al. investigated the antidepressant effect of YZ ethanol extract based on the tail suspension test (TST) and forced swimming test (FST), which are the ease-of-use and widely-accepted models for estimating antidepressant activities in mice. The results indicated that YZ-50 fraction at a dose of 200 mg/kg was able to significantly decrease the immobility time in TST. Furthermore, YZ-50 possessed ability to inhibit corticosterone-induced injury of human neuroblastoma SH-SY5Y cells. What is more, DISS (**123**) and tenuifoliside A (**44**), two major compounds of YZ-50 fraction, showed effective protective response to the lesion in SY5Y cells [53]. The antidepressant-like effect of DISS at the doses of 5, 10, and 20 mg/kg was also tested in chronically mild stressed rats. DISS was able to exhibit antidepressant activity by upregulating the expression of noradrenergic-regulated plasticity genes including cell adhesion molecule L1, brain-derived neurotrophic factor, laminin, and cAMP response element binding protein factor in hippocampus [98]. DISS improved the reward reaction by increasing sucrose intake and obviously decreased the levels of serum cortisol, adrenocorticotrophic hormone, and corticotropin-releasing factor. Further, DISS played an enhanced role in the expression of mineralocorticoid receptor, together with glucocorticoid receptor mRNA [99].

### 3.3. Cytotoxic and Antineoplastic Activities

Smilasides A–F and P, smiglasides A and B, smilaside P, and helonioside A were isolated from the* Smilax*,* Trillium*, and* Paris* genera. Kuo et al. obtained smilasides A–F (**51**–**56**) and evaluated their cytotoxicity against human tumor cell lines comprising human oral epithelium carcinoma (KB), human cervical carcinoma (Hela), human colon tumor (DLD-1), human breast adenocarcinoma (MCF-7), human lung carcinoma (A-549), and human medulloblastoma (Med) cells by MTT assay. Experimental data indicated that all but smilaside C showed cytotoxicity against three to six human tumor cell lines (ED_50_ = 5.1–13.0 *μ*g/mL), and smilasides D–F (ED_50_ = 2.7–5.0 *μ*g/mL) displayed strong cytotoxic activities against DLD-1 cells [46]. Wang et al. reported the antitumor constituents of* Smilax riparia*, including smiglasides A (**48**) and B (**49**), 2,6-di-acetyl-3′,6′-di-feruloylsucrose (**73**), helonioside B (**40**), and smilaside P (**65**). Only smiglasides A and B, and smilaside P exhibited cytotoxicity against human tumor cell lines with different inhibitory concentrations comparing with cisplatin and paclitaxel as positive controls [47]. Helonioside A (**39**) exhibited higher cytotoxicity with the increase of concentration (0.1–100 *μ*g/mL) [45]. Tatarisides A–G (**45**,** 119**–**121**,** 46**,** 122**,** 47**) and diboside A (**116**) from the* Fagopyrum* genus exerted cytotoxic activities against different human cell lines, and the cytotoxicity of tatariside C was the most remarkable with the IC_50_ values ranging from 6.44 to 7.49 *μ*g/mL [57].

1′,2,3,6-*O*-Tetra-acetyl-3′-*O*-*cis*-feruloylsucrose (**95**) from the* Sparganium* plants exhibited extremely weak cytotoxicity against the growth of mice Lung Adenocarcinoma 795 cell lines with an IC_50_ value of 116 *μ*g/mL [66]. SnS-2, oligosaccharides mixture, including raffinose (**3**), stachyose (**19**), and verbascose (**21**) from the roots of* Scrophularia ningpoensis*, had antitumor activity against the growth of Lewis pulmonary carcinoma cells transplanted into mice [18].

Disaccharide esters and oligosaccharides mixture from the Liliaceae, Polygonaceae, Sparganiaceae, and Scrpophulariaceae families showed effective cytotoxic and antineoplastic activities. The study results indicated that feruloyl and acetyl groups play an important role in mediating cytotoxicity, which seems to be related to the substitution position of feruloyl groups. The feruloyl groups at C-6 or C-1′ are vital for cytotoxicity. In addition, the increased number of acetyl groups could induce higher tumoricidal activity.

### 3.4. Anti-Inflammatory Activity

Inflammation, an important basic pathological process, is a defense response of biopsy with vascular system to damage stimuli such as pathogens, impaired cells and tissues, and physical and chemical factors. However, if the process of inflammatory response cannot end normally when cell debris and pathogens were cleared, the biological defence response will become causative factor and bring about many diseases, such as diabetes, cardiovascular diseases, metabolic syndrome, and cancer [100, 101].

Tenuifoliside A (**44**) from the* Polygala* genus exhibited strong anti-inflammatory effect not only by suppressing the production of NO, but also by reducing the production of iNOS, prostaglandin E2, cyclooxygenase-2, and proinflammatory cytokines through the inhibition of the mitogen-activated protein kinases pathway and NF-*κ*B pathway [102]. The anti-inflammatory activities of quiquesetinerviusides D (**108**) and E (**109**) from the* Calamus* genus were evaluated in RAW 264.7 cells. Both of them showed significant inhibitory effects against the production of LPS-stimulated NO with the IC_50_ values of 9.0–29.5 *μ*M [70].

Six disaccharide fatty acid esters (**132**–**137**) were isolated from the* Astragalus* and* Equisetum* genera. The anti-inflammatory effects of these isolated compounds have also been documented. The activation of NF-*κ*B could upregulate the expression of proinflammatory cytokines inducible nitric oxide synthase (iNOS) and tumor necrosis factor alpha (TNF-*α*). The NF-*κ*B inhibitory activities of compounds** 132**–**137** were tested in HepG2 cells stimulated with TNF-*α*. All of these compounds could significantly restrain TNF-*α*-induced NF-*κ*B transcriptional activities with the IC_50_ values of 4.4–24.7 *μ*M. Li et al. pointed out that olefinic bonds and the length of the fatty acid moieties contributed to the NF-*κ*B inhibitory activity. Furthermore, the inhibition increased significantly with the increase of the number of olefinic bonds on the aliphatic moiety [77]. These results may provide a scientific basis for the development of new anti-inflammatory agents.

### 3.5. Neuroprotective and Cerebral Protective Activities

As we all know, glutamate works as a major excitatory amino acid neurotransmitter in the mammalian central nervous system and plays a crucial role in several physiological processes [103]. However, the accumulation of glutamate induces diverse acute and chronic neurodegenerative diseases, such as epilepsy, ischemic stroke, and Parkinson's disease, as well as Alzheimer's disease [104]. DISS (**123**) isolated from the* Polygala* genus exhibited neuroprotective effect against glutamate-induced SH-SY5Y neuronal cell damage. The* in vitro* test demonstrated that DISS (0.6, 6 and 60 *μ*mol/L) played a critical role in increasing cell viability, controlling lactate dehydrogenase and attenuated apoptosis ranging from 1.95% to 2.58% [105].

Tenuifoliside B (**102**) from the* Polygala* genus was able to significantly shorten the coma time of KCN-induced anoxia mice at the doses of 3 and 10 mg/kg, and it played an important role in ameliorating the scopolamine-induced impairment of performance in passive avoidance task in rats and enhancing the tremors induced by oxotremorine in mice. These results together demonstrated that tenuifoliside B possessed cognitive improving and cerebral protective effects [56].

### 3.6. Antidiabetic Activity

Diabetes mellitus, a chronic debilitating metabolic disease, is characterized by high blood glucose content and comprises three types termed type I, type II, and gestational diabetes [106]. Stachyose (**19**) extract (a part) from* Rehmannia glutinosa* obviously exhibited the activity of downregulating fasting plasma glucose level and partially keeping from hyperglycemia induced by adrenaline and glucose without obvious dose-dependent effect. Other than that,* in vivo* tests in rats induced by alloxan revealed that stachyose extract at the dose of 200 mg/kg significantly decreased blood-sugar level [15].

Diboside A, lapathosides C and D, vanicosides A and B, and hydropiperoside were isolated from the* Fagopyrum*,* Polygonum*, and* Persicaria* genera belonging to the Polygonaceae family. Diboside A (**116**) could potentially inhibit *α*-amylase activity with an IC_50_ of 26.9 *μ*M and thus retard the starch digestion rate, which is helpful for diabetic individuals in controlling blood sugar level [107]. Lapathoside D (**38**) exerted stronger activity of *α*-glucosidase inhibition with an IC_50_ value of 0.113 mM than acarbose which was chosen as a positive drug for the treatment of type II diabetes [43]. Vanicoside B (**111**) was reported to have higher *β*-glucosidase inhibitory activity with an IC_50_ of 50.5 *μ*M than vanicoside A (**110**) with an IC_50_ of 59.9 *μ*M because of the acetyl moiety of the latter possibly decreasing inhibitory activity of vanicoside A [71].

Fujimoto et al. investigated the inhibitory effects of mumeoses F–O (**83**–**92**) from the* Prunus* genus on aldose reductase and discovered that caffeoyl groups are crucial for the inhibitory effect on aldose reductase. And thus, mumeoses F, G, H, J, K, L, M and N (IC_50_ = 22–77 *μ*M), with a coumaroyl group and acetyl groups, inhibited moderately aldose reductase from reducing glucose to sorbitol, which is associated with the chronic complications of diabetes [63, 64].

### 3.7. Elicitors and Regulators

Oligosaccharides are quite propitious for encoding biological information because of diverse monosaccharide units and complex molecular structures and they are therefore first described as biological signals in plants [108]. Oligosaccharides from the cell wall fragments of plants and fungi are powerful signal molecules, such as the elicitors of plant defence response and the regulators of plant growth, and they are capable of exerting biological activities at exceedingly low concentrations [109]. Heptasaccharide (HS) (**15**) and octasaccharide (OS) (**16**) isolated from the* Paris* genus possessed plant growth-regulatory activities [29, 30]. The two oligosaccharides significantly promoted the proliferation of* Paris polyphylla* var.* yunnanensis* roots at the doses of 2.5–20 mg/L. The octasaccharide had the most obvious effect on the growth of* Panax japonicus* var.* major* hairy roots at a dose of 30 mg/L, while the other had the most positive effect on saponin accumulation of* Panax japonicus* var.* major *hairy roots at a dose of 10 mg/L [29]. Similarly, Zhou et al. evaluated the stimulating effects of HS and OS on the root growth and saponin production of* Panax ginseng* hairy roots, which were induced from the plant roots infected with* Agrobacterium rhizogenes* strain A4. The results showed that there was a maximum effect on the hairy roots growth and saponin accumulation on day 10. Compared with control group, the root biomass dry weight was increased by more than 1.7-fold while the total saponin content of roots increased by more than 1-fold when these two oligosaccharides were added to the hairy root at a dose of 30 mg/L [30]. The above data illustrate that HS and OS could serve as the plant growth-regulators not only in their original species but also in others.

### 3.8. Immunopotentiating Activity

Macrophages are important targets of investigations on cytophagy, cellular immunity, and molecular immunology. Therefore, they are deemed to play a vital role in host defense comprising phagocytosis, proteolytic processing, pathogenic agent, apoptosis, cytokines production, and foreign antigens presentation [110]. The water-extracted oligosaccharides from* Panax ginseng* (WGOS) exhibited better immunopotentiating activity by increasing phagocytic function of macrophages and promoting NO, TNF-*α* and reactive oxygen species production [110]. In addition, Wan et al. have obtained malto-oligosaccharides (**17**, *n* = 3~8) and three oligosaccharides (**2**,** 13**,** 14**) from the* Panax ginseng *roots. The* in vitro* bioassay pointed out that WGOS could serve as efficacious stimulators of B and T lymphocytes [28]. These studies provided enlightenment that the mixture of oligosaccharides from Chinese herbal medicine exhibits significant effect on immune system.

### 3.9. Others

Acetylcholinesterase (AChE) inhibitors show good therapeutic effects on myasthenia gravis, glaucoma, and Alzheimer's disease through reversible enzyme inhibition so as to increase the accumulation of acetylcholine in the synapse and then promote and prolong the function of acetylcholine. Vanicoside B (**111**) showed AChE inhibitory activity with an IC_50_ of 0.062 mM, while hydropiperoside (**42**), and lapathosides C (**115**) and D (**38**) just exhibited weak enzyme inhibitory activity [43].

Wang et al. has explored low molecular mass carbohydrate polymer from* Panax ginseng* roots and obtained 30% ethanol elution (PGO) which included peptides and oligosaccharides (**17**, *n* = 0~5) identified as maltose, maltotriose, maltotetraose, maltopentaose, maltohexaose, and maltoheptaose. Pharmacological experiments revealed that PGO could significantly enhance the memory in scopolamine-induced memory deficit rats [27].

Cistanoside F (**129**) and kankanose (**150**) were isolated from the* Cistanche*,* Paulownia*, and* Acanthus* genera. Pharmacological experiments showed that cistanoside F and kankanose significantly exhibited vasorelaxant effects on the noradrenaline-induced contraction of thoracic aorta from rats [80].

## 4. Conclusion

Traditional Chinese medicine from natural kingdom plays an indelible role in the treatment of human diseases, and it has aroused the attention of those who have engaged in medicinal pharmaceutical chemistry. Therefore, scientists have made great contributions day after day to investigate the valid chemicals from traditional Chinese medicines. In the past decades, about 193 oligosaccharides and their esters have been identified from traditional Chinese medicinal plants. On the one hand, only a few oligosaccharides and their mixtures were investigated and just exhibited antidepressant, antineoplastic, antidiabetic, plant growth-regulatory, immunopotentiating, and enhanced memory activities. More exploratory work is still needed to excavate biological and pharmacological activities of oligosaccharides. On the other hand, oligosaccharide esters exhibited multi-advantageous activities. Bioassays have revealed that antioxidant, cytotoxic, antineoplastic, and anti-inflammatory activities are the most notable bioactivities. Of course, to search for the natural products with these activities is a hotpot in the contemporary drug research. Oligosaccharide esters provide a vast treasure trove for medical researchers. After considering the current studies, it should be taken as future directions to make more mechanism of action studies and clinical trials to further evaluate its potential as new drugs. Moreover, the structure-activity relationships discussed in this review will provide reference information for further exploring their relationships and continually discovering the new bioactive oligosaccharide esters.

## Figures and Tables

**Scheme 1:**
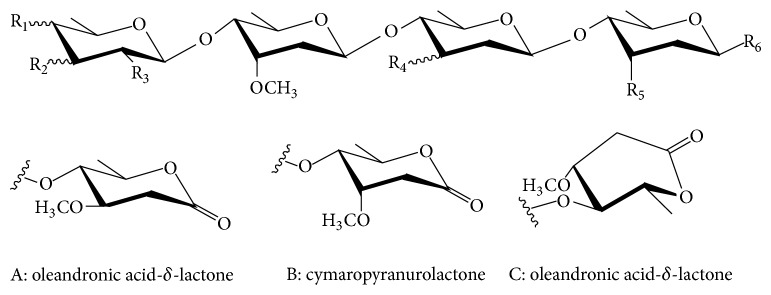


**Scheme 2:**
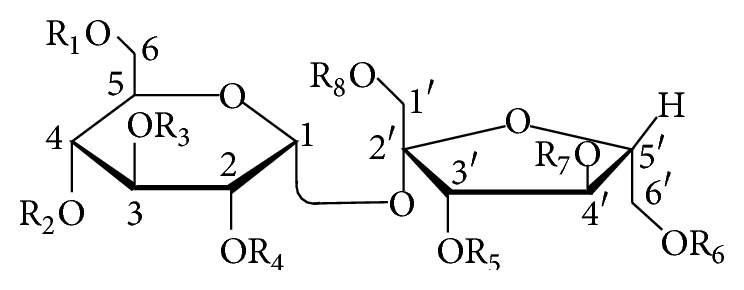


**Scheme 3:**
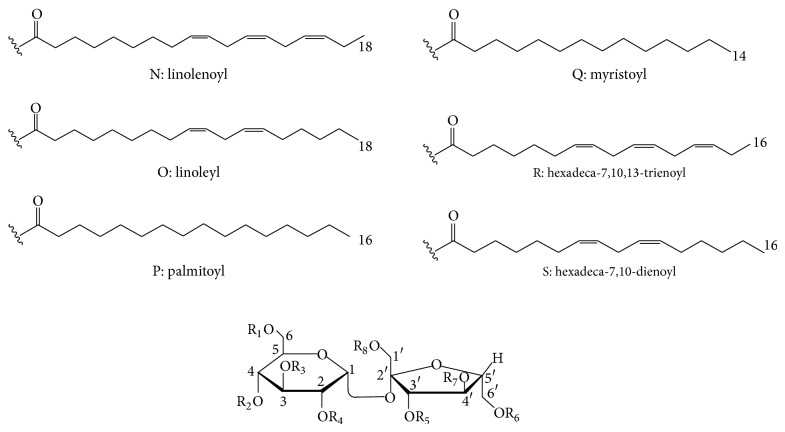


**Scheme 4:**
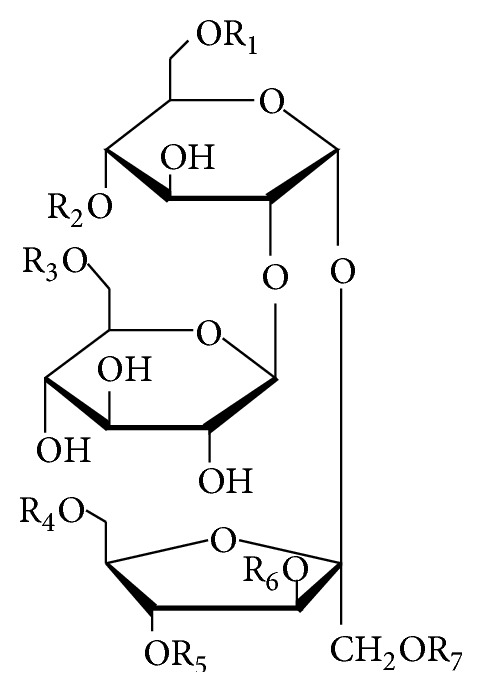


**Scheme 5:**
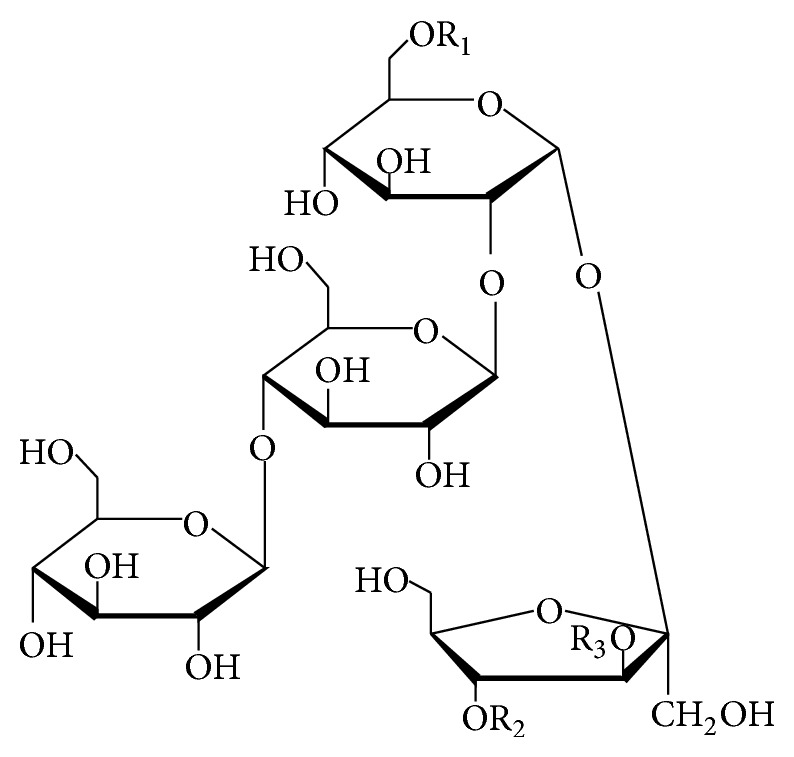


**Scheme 6:**
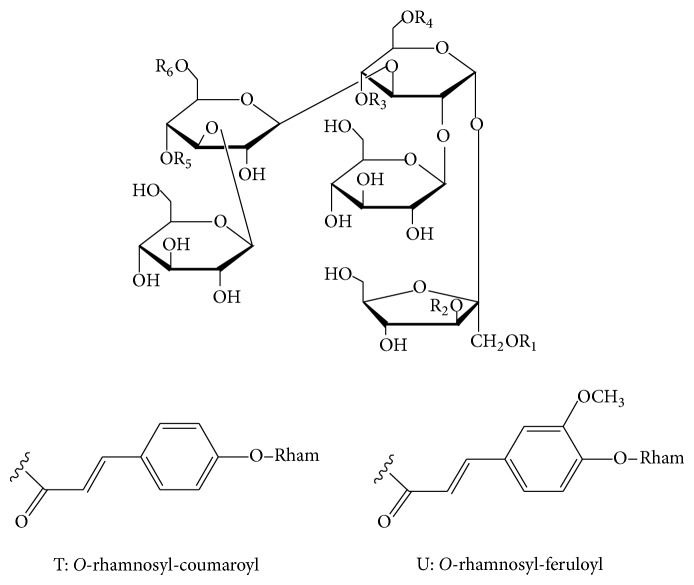


**Table 1:** Oligosaccharides.

No.	Name	R_1_	R_2_	R_3_	R_4_	R_5_	R_6_	Source	Parts	Reference

**22**	Perifosaccharide A	OH(e)	OCH_3_(e)	H	OH(e)	OH	A	*Periploca forrestii *	Roots	[32]

**23**	Perifosaccharide B	OH(e)	OCH_3_(a)	H	OH(e)	OH	A	*Periploca forrestii *	Roots	[32]

**24**	Perifosaccharide C	OH(e)	OCH_3_(e)	H	OH(e)	OCH_3_	A	*Periploca forrestii *	Roots	[32]

**25**	Perifosaccharide D	OAc(e)	OCH_3_(e)	H	OH(e)	OH	A	*Periploca forrestii *	Roots	[32]

**26**	Perisaccharide A	OH(a)	OCH_3_(e)	OAc	OH(e)	OCH_3_	A	*Periploca sepium *	Root barks	[33]

**27**	Perisaccharide B	OAc(e)	OCH_3_(a)	H	OH(e)	OH	A	*Periploca sepium* *Periploca calophylla *	Root barks	[33, 34]

**28**	Perisaccharide C	OH(a)	OCH_3_(e)	OAc	OCH_3_(a)	OH	A	*Periploca sepium *	Root barks	[33]

**29**	Perisaccharide D	OAc(e)	OCH_3_(a)	H	OH(e)	OCH_3_	A	*Periploca calophylla *	Stems	[34]

**30**	Perisesaccharide B	OH(a)	OCH_3_(e)	OAc	OH(e)	OCH_3_	C	*Periploca sepium *	Root barks	[35]

**31**	Perisesaccharide C	OH(a)	OCH_3_(e)	OH	OCH_3_(a)	OCH_3_	C	*Periploca sepium *	Root barks	[35]

**32**	Perisesaccharide D	OH(a)	OCH_3_(e)	OH	OCH_3_(a)	OH	C	*Periploca sepium *	Root barks	[35]

**33**	Perisesaccharide E	OH(a)	OCH_3_(e)	OAc	OH(e)	OH	C	*Periploca sepium *	Root barks	[35]

**34**	Cymaropyranurolactone 4-*O*-*β*-D-digitalopyranosyl-(1 → 4)-*O*-*β*-D-cymaropyranosyl-(1 → 4)-*O*-*β*-D-oleandropyranosyl-(1 → 4)-*O*-*β*-D-cymaropyranoside	OH(a)	OCH_3_(e)	OH	OCH_3_(e)	OCH_3_	B	*Parabarium huaitingii *	Barks	[36]

See Scheme 1.

**Table 2:** Phenylpropanoid-derived disaccharide esters.

Number	Name	R_1_	R_2_	R_3_	R_4_	R_5_	R_6_	R_7_	R_8_	Source	Parts	Reference
**35**	Sibiricose A_6_	H	H	H	H	K	H	H	H	*Polygala tricornis* *Polygala tenuifolia *	Roots Root barks	[37–40]
**36**	3′-*O*-Feruloyl sucrose/Sibiricose A_5_	H	H	H	H	I	H	H	H	*Polygala tenuifolia * *Trillium kamtschaticum *	Roots Underground parts	[38–42]
**37**	Glomeratose A	H	H	H	H	L	H	H	H	*Polygala tricornis *	Roots	[37]
**38**	Lapathoside D	H	H	H	H	G	G	H	H	*Polygonum sachalinense *	Stems	[43]
**39**	Helonioside A	H	H	H	H	I	I	H	H	*Trillium kamtschaticum* *Smilax bracteata* *Paris polyphylla * var*. yunnanensis *	Underground parts Aerial parts Roots	[41, 44, 45]
**40**	Helonioside B	Ac	H	H	H	I	I	H	H	*Smilax bracteata* * Smilax china* *Smilax riparia* *Heterosmilax erythrantha *	Aerial parts Stems Roots and rhizomes	[44, 46–48]
**41**	Parispolyside F	H	H	H	H	G	I	H	H	*Paris Polyphylla * var*. yunnanensis *	Rhizomes	[49, 50]
**42**	Hydropiperoside	H	H	H	H	G	G	H	G	*Polygonum sachalinense* *Polygonum cuspidatum * *Persicaria hydropiper *	Stems Leaves	[43, 51, 52]
**43**	Tricornose B	D	Ac	H	H	L	H	H	H	*Polygala tricornis *	Roots	[37]
**44**	Tenuifoliside A	F	H	H	H	L	H	H	H	*Polygala tenuifolia* * Polygala hongkongensis* * Polygala sibirica *	Roots Whole plants Aerial parts	[38, 40, 42, 53–56]
**45**	Tatariside A	Ac	H	H	Ac	G	G	H	Ac	*Fagopyrum tataricum *	Roots	[57]
**46**	Tatariside E	H	H	H	Ac	G	G	H	Ac	*Fagopyrum tataricum *	Roots	[57]
**47**	Tatariside G	H	H	H	H	I	G	H	G	*Fagopyrum tataricum *	Roots	[57]
**48**	Smiglaside A	Ac	Ac	H	Ac	I	I	H	I	*Smilax riparia *	Roots and rhizomes	[47]
**49**	Smiglaside B	Ac	H	H	Ac	I	I	H	I	*Smilax riparia *	Roots and rhizomes	[47]
**50**	Smiglaside E	Ac	H	H	Ac	I	I	H	G	*Smilax china *	Stems	[46]
**51**	Smilaside A	Ac	Ac	H	H	I	I	H	H	*Smilax china *	Stems	[46]
**52**	Smilaside B	H	H	H	Ac	I	I	H	H	*Smilax china *	Stems	[46]
**53**	Smilaside C	H	H	H	H	I	I	H	G	*Smilax china*	Stems	[46]
**54**	Smilaside D	H	H	H	H	I	I	Ac	G	*Smilax china *	Stems	[46]
**55**	Smilaside E	Ac	H	H	H	I	I	H	G	*Smilax bracteata* *Smilax china *	Aerial parts Stems	[44, 46]
**56**	Smilaside F	Ac	H	H	Ac	G	I	H	G	*Smilax china *	Stems	[46]
**57**	Smilaside G	H	H	H	H	G	I	H	G	*Smilax bracteata * * Smilacis glabrae *	Aerial parts Rhizomes	[44, 58]
**58**	Smilaside H	H	H	H	Ac	G	I	H	G	*Smilax bracteata *	Aerial parts	[44]
**59**	Smilaside I	Ac	H	H	H	G	I	H	G	*Smilax bracteata *	Aerial parts	[44]
**60**	Smilaside J	H	H	H	H	G	I	H	I	*Smilax bracteata * * Smilacis glabrae *	Aerial parts Rhizomes	[44, 58]
**61**	Smilaside K	H	H	H	Ac	I	I	H	G	*Smilax bracteata *	Aerial parts	[44]
**62**	Smilaside L	H	H	H	H	I	I	H	I	*Smilax bracteata* *Smilacis glabrae *	Aerial parts Rhizomes	[44, 58]
**63**	Smilaside M	Ac	H	H	Ac	*Cis*-feruloyl	I	H	H	*Smilax riparia *	Roots and rhizomes	[59]
**64**	Smilaside N	Ac	H	H	Ac	I	*Cis*-feruloyl	H	H	*Smilax riparia *	Roots and rhizomes	[59]
**65**	Smilaside P	H	H	H	Ac	I	I	H	I	*Smilax riparia *	Roots and rhizomes	[47]
**66**	3′,4′,6′-*O*-Tri-feruloylsucrose	H	H	H	H	I	I	I	H	*Smilax riparia *	Rhizomes and roots	[60]
**67**	6′-*O*-Coumaroylsucrose	H	H	H	H	H	G	H	H	*Bidens parviflora *	Whole plants	[61]
**68**	1′-*O*-Coumaroyl-6′-*O*-feruloylsucrose	H	H	H	H	H	I	H	G	*Smilax bracteata *	Aerial parts	[44]
**69**	4-*O*-Benzoyl-3′-3,4,5-trimethoxycinnamoylsucrose	H	D	H	H	L	H	H	H	*Polygala tricornis *	Roots	[37]
**70**	6-*O*-Benzoyl-3′-*O*-3,4,5-trimethoxycinnamoylsucrose	D	H	H	H	L	H	H	H	*Polygala tricornis *	Roots	[37]
**71**	6-*O*-Benzoyl-3′-*O*-sinapoylsucrose	D	H	H	H	K	H	H	H	*Polygala tricornis *	Roots	[37]
**72**	6-*O*-*p*-Methoxybenzoyl-3′-*O*-3,4,5-trimethoxycinnamoylsucrose	E	H	H	H	L	H	H	H	*Polygala tenuifolia *	Roots	[56]
**73**	2,6-Di-acetyl-3′,6′-di-feruloylsucrose	Ac	H	H	Ac	I	I	H	H	*Smilax china* *Smilax riparia* *Heterosmilax erythrantha *	Stems Roots and rhizomes Aerial parts	[46–48, 59]
**74**	2,6-Di-acetyl-3′-*O*-*cis*-feruloy-6′-trans-feruloylsucrose	Ac	H	H	Ac	*Cis*-feruloy	I	H	H	*Smilax riparia *	Roots and rhizomes	[59]
**75**	2,6-Di-acetyl-3′-*O*-*trans*-feruloy-6′-*cis*-feruloylsucrose	Ac	H	H	Ac	I	*Cis*-feruloyl	H	H	*Smilax riparia *	Roots and rhizomes	[59]
**76**	Regaloside A	Ac	H	H	H	I	H	H	H	*Trillium kamtschaticum *	Underground parts	[41]
**77**	Tricornose A	Ac	H	H	H	L	H	H	H	*Polygala tricornis *	Roots	[37]
**78**	Mumeose A	H	H	H	Ac	G	H	H	H	*Prunus mume *	Flower buds	[62, 63]
**79**	Mumeose B	Ac	H	Ac	H	G	H	H	H	*Prunus mume *	Flower buds	[62, 63]
**80**	Mumeose C	Ac	H	Ac	Ac	G	H	H	H	*Prunus mume *	Flower buds	[62, 63]
**81**	Mumeose D	Ac	Ac	Ac	Ac	G	H	H	Ac	*Prunus mume *	Flower buds	[62, 63]
**82**	Mumeose E	Ac	Ac	Ac	Ac	*Cis*-coumaroyl	H	H	Ac	*Prunus mume *	Flower buds	[62, 63]
**83**	Mumeose F	Ac	Ac	Ac	H	G	H	H	H	*Prunus mume *	Flower buds	[63]
**84**	Mumeose G	Ac	H	Ac	H	G	H	Ac	H	*Prunus mume *	Flower buds	[63]
**85**	Mumeose H	H	H	Ac	Ac	G	H	Ac	H	*Prunus mume *	Flower buds	[63]
**86**	Mumeose I	Ac	Ac	Ac	H	G	Ac	H	H	*Prunus mume *	Flower buds	[63]
**87**	Mumeose J	Ac	Ac	Ac	Ac	G	H	Ac	Ac	*Prunus mume *	Flower buds	[63]
**88**	Mumeose K	H	H	Ac	Ac	G	H	H	H	*Prunus mume *	Flower buds	[64]
**89**	Mumeose L	Ac	H	Ac	Ac	G	H	Ac	H	*Prunus mume *	Flower buds	[64]
**90**	Mumeose M	Ac	Ac	Ac	H	G	H	Ac	Ac	*Prunus mume *	Flower buds	[64]
**91**	Mumeose N	Ac	Ac	Ac	H	G	Ac	Ac	H	*Prunus mume *	Flower buds	[64]
**92**	Mumeose O	Ac	H	Ac	Ac	G	H	Ac	Ac	*Prunus mume *	Flower buds	[64]
**93**	1′,2,3,4,6-*O*-Penta-acetyl-3′-*O*-*trans*-coumaroylsucrose	Ac	Ac	Ac	Ac	G	H	H	Ac	*Musella lasiocarpa *	Aerial parts	[65]
**94**	1′,2,3,4,6-*O*-Penta-acetyl-3′-*O*-*cis*-coumaroylsucrose	Ac	Ac	Ac	Ac	*Cis*-coumaroyl	H	H	Ac	*Musella lasiocarpa *	Aerial parts	[65]
**95**	1′,2,3,6-*O*-Tetra-acetyl-3′-*O*-*cis*-feruloylsucrose	Ac	H	Ac	Ac	*Cis*-feruloyl	H	H	Ac	*Sparganium stoloniferum *	Rhizomes	[66]
**96**	1′,2,4,6-O-Tetra-acetyl-3′-O-*trans*-feruloylsucrose	Ac	Ac	H	Ac	I	H	H	Ac	*Sparganium stoloniferum *	Rhizomes	[66]
**97**	1′,2,3,6-O-Tetra-acetyl-3′-O-*trans*-feruloylsucrose	Ac	H	Ac	Ac	I	H	H	Ac	*Sparganium stoloniferum *	Rhizomes	[67]
**98**	Sibirioside A	M	H	H	H	H	H	H	H	*Scrophularia ningpoensis *	Roots	[68]
**99**	Sibricose A_1_	K	H	H	H	H	H	H	H	*Cynanchum amplexicaule *	Roots	[69]
**100**	6-*O*-Caffeoylsucrose	J	H	H	H	H	H	H	H	*Scrophularia ningpoensis *	Roots	[68]
**101**	Acretoside	I	H	H	H	H	H	H	H	*Scrophularia ningpoensis *	Roots	[68]
**102**	Tenuifoliside B	F	H	H	H	K	H	H	H	*Polygala tenuifolia *	Roots	[42, 56]
**103**	Tenuifoliside C	K	H	H	H	L	H	H	H	*Polygala tricornis* *Polygala tenuifolia *	Roots	[37, 42, 56]
**104**	Heterosmilaside	H	H	I	H	H	I	H	H	*Heterosmilax erythrantha *	Aerial parts	[48]
**105**	Quiquesetinerviuside A	H	I	H	H	I	I	H	H	*Calamus quiquesetinervius *	Stems	[70]
**106**	Quiquesetinerviuside B	Ac	I	H	H	I	I	H	H	*Calamus quiquesetinervius *	Stems	[70]
**107**	Quiquesetinerviuside C	H	I	H	Ac	I	I	H	H	*Calamus quiquesetinervius *	Stems	[70]
**108**	Quiquesetinerviuside D	Ac	G	H	H	I	I	H	H	*Calamus quiquesetinervius *	Stems	[70]
**109**	Quiquesetinerviuside E	H	G	H	Ac	I	I	H	H	*Calamus quiquesetinervius *	Stems	[70]
**110**	Vanicoside A	I	H	H	Ac	G	G	H	G	*Polygonum sachalinensis * *Polygonum cuspidatum* *Polygonum hydropiper *	Stems Leaves Rhizomes	[51, 52, 71, 72]
**111**	Vanicoside B	I	H	H	H	G	G	H	G	*Polygonum sachalinensis * *Polygonum cuspidatum * *Persicaria hydropiper *	Stems Leaves Rhizomes	[43, 51, 52, 71, 72]
**112**	Vanicoside D	G	H	H	H	G	G	H	G	*Persicaria hydropiper *	Leaves	[51]
**113**	Vanicoside E	I	Ac	H	Ac	G	G	H	G	*Polygonum hydropiper *	Stems and leaves	[72]
**114**	Lapathoside A	I	H	H	H	G	G	H	I	*Polygonum sachalinensis* *Polygonum cuspidatum * *Fagopyrum dibotrys *	Stems Rhizomes	[51, 73]
**115**	Lapathoside C	I	H	H	H	G	G	H	H	*Polygonum sachalinensis* *Polygonum cuspidatum *	Stems	[43, 52]
**116**	Diboside A	G	H	H	H	G	I	H	G	*Fagopyrum tataricum* *Fagopyrum dibotrys *	Roots Rhizomes	[57]
**117**	Hidropiperoside A	I	H	H	H	H	G	H	G	*Polygonum hydropiper *	Stems and leaves	[72]
**118**	Hidropiperoside B	I	H	H	Ac	G	G	H	I	*Polygonum hydropiper *	Stems and leaves	[72]
**119**	Tatariside B	I	H	H	Ac	G	G	H	Ac	*Fagopyrum tataricum *	Roots	[57]
**120**	Tatariside C	I	Ac	H	Ac	G	G	H	Ac	*Fagopyrum tataricum *	Roots	[57]
**121**	Tatariside D	G	H	H	Ac	G	I	H	H	*Fagopyrum tataricum *	Roots	[57]
**122**	Tatariside F	G	H	H	H	I	I	H	G	*Fagopyrum tataricum *	Roots	[57]
**123**	3′,6-*O*-Di-sinapoylsucrose	K	H	H	H	K	H	H	H	*Polygala tricornis* *Polygala hongkongensis* *Polygala sibirica* *Polygala tenuifolia* *Cynanchum amplexicaule *	Roots Root barks Whole plants Aerial parts	[37–39, 41, 53–56, 69, 74]
**124**	6,6′-*O*-Di-coumaroylsucrose	G	H	H	H	H	G	H	H	*Bidens parviflora *	Whole plants	[61]
**125**	1′,3′,6′-*O*-Tri-coumaroyl-6-feruloylsucrose	I	H	H	H	G	G	H	G	*Fagopyrum tataricum *	Roots	[75, 76]
**126**	3′,6′-*O*-Di-coumaroyl-1′,6-*O*-di-feruloylsucrose	I	H	H	H	G	G	H	I	*Fagopyrum tataricum *	Roots	[75, 76]
**127**	3′-*O*-Coumaroyl-1′,6′,6-*O*-tri-feruloylsucrose	I	H	H	H	I	G	H	I	*Fagopyrum tataricum *	Roots	[75, 76]
**128**	1′,3′,6′,6-*O*-Tetra-feruloylsucrose	I	H	H	H	I	I	H	I	*Fagopyrum tataricum *	Roots	[75, 76]

****See Scheme 2.

**Table 3:** Fatty acid-derived disaccharide esters.

Number	Name	R_1_	R_2_	Source	Parts	Reference

**132**	6′-*O*-Linoleylsucrose	H	O	*Astragalus membranaceus *	Roots	[77]
**133**	6′-*O*-Palmitoylsucrose	H	P	*Astragalus membranaceus *	Roots	[77]
**134**	6-*O*-Palmitoylsucrose	P	H	*Astragalus membranaceus *	Roots	[77]
**135**	6′-*O*-Linolenoylsucrose	H	N	*Astragalus membranaceus* *Equisetum hiemale *	Roots Aerial parts	[77, 78]
**136**	6-*O*-Linoleylsucrose	O	H	*Astragalus membranaceus *	Roots	[77]
**137**	6-*O*-Myristoylsucrose	Q	H	*Astragalus membranaceus *	Roots	[77]
**138**	6-*O*-[(7*Z*,10*Z*,13*Z*)-Hexadeca-7,10,13-trienoyl]sucrose	H	R	*Equisetum hiemale *	Aerial parts	[78]
**139**	6-*O*-[(7*Z*,10*Z*)-Hexadeca-7,10-dienoyl]sucrose	H	S	*Equisetum hiemale *	Aerial parts	[78]

See Scheme 3.

**Table 4:** Phenylpropanoid-derived trisaccharide esters.

Number	Name	R_1_	R_2_	R_3_	R_4_	R_5_	R_6_	R_7_	Source	Parts	Reference

**146**	Tricornose C	K	H	H	H	H	L	H	*Polygala tricornis *	Roots	[37]
**147**	Tricornose D	K	H	H	H	H	K	H	*Polygala tricornis *	Roots	[37]
**148**	Tricornose E	K	H	H	H	K	L	H	*Polygala tricornis *	Roots	[37]
**149**	Tricornose F	K	H	H	H	I	L	H	*Polygala tricornis *	Roots	[37]

See Scheme 4.

**Table 5:** Phenylpropanoid-derived tetrasaccharide esters.

Number	Name	R_1_	R_2_	R_3_	Source	Parts	Reference

**154**	Tricornose G	K	H	K	*Polygala tricornis *	Roots	[37]
**155**	Tricornose H	K	K	K	*Polygala tricornis *	Roots	[37]
**156**	Tricornose I	K	K	L	*Polygala tricornis *	Roots	[37]
**157**	Tricornose J	K	I	L	*Polygala tricornis *	Roots	[37]
**158**	Tricornose K	K	I	K	*Polygala tricornis *	Roots	[37]
**159**	Tricornose L	K	G	K	*Polygala tricornis *	Roots	[37]

See Scheme 5.

**Table 6:** Phenylpropanoid-derived pentasaccharide esters.

Number	Name	R_1_	R_2_	R_3_	R_4_	R_5_	R_6_	Source	Parts	Reference

**160**	Tenuifoliose A	G	D	I	Ac	Ac	Ac	*Polygala tenuifolia *	Roots	[38, 42]
**161**	Tenuifoliose B	G	D	I	H	Ac	Ac	*Polygala tenuifolia *	Roots	[42]
**162**	Tenuifoliose C	G	D	I	H	H	H	*Polygala tenuifolia *	Roots	[42]
**163**	Tenuifoliose F	G	D	U	Ac	Ac	Ac	*Polygala tenuifolia *	Roots	[42]
**164**	Tenuifoliose G	G	D	U	Ac	H	Ac	*Polygala tenuifolia *	Roots	[42]
**165**	Tenuifoliose H	G	D	G	Ac	Ac	Ac	*Polygala tenuifolia *	Roots	[38, 42, 79]
**166**	Tenuifoliose I	G	D	G	Ac	H	Ac	*Polygala tenuifolia *	Roots	[42, 79]
**167**	Tenuifoliose J	G	D	G	H	Ac	Ac	*Polygala tenuifolia *	Roots	[42]
**168**	Tenuifoliose K	G	D	G	H	H	Ac	*Polygala tenuifolia *	Roots	[42]
**169**	Tenuifoliose L	G	D	T	Ac	Ac	Ac	*Polygala tenuifolia *	Roots	[42, 79]
**170**	Tenuifoliose M	G	D	T	Ac	H	Ac	*Polygala tenuifolia *	Roots	[42]
**171**	Tenuifoliose N	I	D	I	Ac	Ac	Ac	*Polygala tenuifolia *	Roots	[42]
**172**	Tenuifoliose O	I	D	I	H	Ac	Ac	*Polygala tenuifolia *	Roots	[42]
**173**	Tenuifoliose P	I	D	I	H	H	Ac	*Polygala tenuifolia *	Roots	[42]
**174**	Tenuifoliose Q	G	D	T	H	Ac	Ac	*Polygala tenuifolia *	Roots	[79]
**175**	Tenuifoliose S	G	D	G	H	H	H	*Polygala tenuifolia *	Roots	[42]
**176**	Tenuifoliose T	G	D	I	H	H	H	*Polygala tenuifolia *	Roots	[42]
**177**	Tenuifoliose V	H	D	I	Ac	Ac	Ac	*Polygala tenuifolia *	Roots	[42]
**178**	Tenuifoliose W	I	D	U	Ac	H	Ac	*Polygala tenuifolia *	Roots	[42]
**179**	Tenuifoliose X	I	D	I	H	H	H	*Polygala tenuifolia *	Roots	[42]

See Scheme 6.

## References

[1] Yang X. D., Zhang L. J., Liang B., Xu L. Z., Yang S. L. (2002). Oligosaccharide esters isolated from plants of Polygalaceae. *Chinese Traditional and Herbal Drugs*.

[2] Ogwuru N., Adamczeski M. (2000). Bioactive natural products derived from *Polygonum* species of plants: their structures and mechanisms of action. *Studies in Natural Products Chemistry*.

[3] Kobayashi S., Miyase T., Noguchi H. (2002). Polyphenolic glycosides and oligosaccharide multiesters from the roots of *Polygala dalmaisiana*. *Journal of Natural Products*.

[4] Takasaki M., Kuroki S., Kozuka M., Konoshima T. (2001). New phenylpropanoid esters of sucrose from *Polygonum lapathifolium*. *Journal of Natural Products*.

[5] Zimmermann M. L., Sneden A. T. (1994). Vanicosides A and B, protein kinase C inhibitors from *Polygonum pensylvanicum*. *Journal of Natural Products*.

[6] Takasaki M., Konoshima T., Kuroki S., Tokuda H., Nishino H. (2001). Cancer chemopreventive activity of phenylpropanoid esters of sucrose, vanicoside B and lapathoside A, from Polygonum lapathifolium. *Cancer Letters*.

[7] Fu Q., Liang T., Li Z. Y. (2013). Separation of carbohydrates using hydrophilic interaction liquid chromatography. *Carbohydrate Research*.

[8] Mrozek M. F., Zhang D., Ben-Amotz D. (2004). Oligosaccharide identification and mixture quantification using Raman spectroscopy and chemometric analysis. *Carbohydrate Research*.

[9] Armstrong G. S., Bendiak B. (2006). High-resolution four-dimensional carbon-correlated ^1^H-^1^H ROESY experiments employing isotags and the filter diagonalization method for effective assignment of glycosidic linkages in oligosaccharides. *Journal of Magnetic Resonance*.

[10] Armstrong G. S., Mandelshtam V. A., Shaka A. J., Bendiak B. (2005). Rapid high-resolution four-dimensional NMR spectroscopy using the filter diagonalization method and its advantages for detailed structural elucidation of oligosaccharides. *Journal of Magnetic Resonance*.

[11] Tseng H.-M., Gattolin S., Pritchard J., Newbury H. J., Barrett D. A. (2009). Analysis of mono-, di- and oligosaccharides by CE using a two-stage derivatization method and LIF detection. *Electrophoresis*.

[12] Li N., Li X., Hou B. L., Meng D. L. (2008). New disaccharoside from *Camptosorus sibiricus* Rupr. *Natural Product Research*.

[13] Zhang Z., Wang D., Zhao Y., Gao H., Hu Y.-H., Hu J.-F. (2009). Fructose-derived carbohydrates from *Alisma orientalis*. *Natural Product Research*.

[14] Yang X., Zhao Y., He N., Croft K. D. (2010). Isolation, characterization, and immunological effects of *α*-galacto-oligosaccharides from a new source, the herb *Lycopus lucidus* Turcz. *Journal of Agricultural and Food Chemistry*.

[15] Zhang R. X., Jia Z. P., Kong L. Y. (2004). Stachyose extract from *Rehmannia glutinosa* Libosch. to lower plasma glucose in normal and diabetic rats by oral administration. *Pharmazie*.

[16] Zhang R., Zhao Y., Sun Y., Lu X., Yang X. (2013). Isolation, characterization, and hepatoprotective effects of the raffinose family oligosaccharides from *Rehmannia glutinosa* libosch. *Journal of Agricultural and Food Chemistry*.

[17] Li H., Song F., Zheng Z., Liu Z., Liu S. (2008). Characterization of saccharides and phenolic acids in the Chinese herb Tanshen by ESI-FT-ICR-MS and HPLC. *Journal of Mass Spectrometry*.

[18] Deng J. E., Zhang J., Chen X. M., Ke W., Tian G. Y. (2004). Studies on the physicochemical properties, structure and antitumor activity of an oligosaccharide homologue SnS-2 from the root of *Scrophularia ningpoensis* Hemsl. *Chinese Journal of Chemistry*.

[19] Chou S. C., Chuang L. M., Lee S. S. (2012). Hypoglycemic constituents of *gynura divaricata* subsp. *formosana*. *Natural Product Communications*.

[20] Feng F., Wang L.-L., Lai X.-P., Li Y.-B., Cao Z.-M., Zhou Y.-J. (2012). Study on oligosaccharides from *Morinda officinalis*. *Journal of Chinese Medicinal Materials*.

[21] Li Y. F., Gong Z. H., Yang M., Zhao Y. M., Luo Z. P. (2003). Inhibition of the oligosaccharides extracted from *Morinda officinalis*, a Chinese traditional herbal medicine, on the corticosterone induced apoptosis in PC12 cells. *Life Sciences*.

[22] Li Y.-F., Liu Y.-Q., Yang M. (2004). The cytoprotective effect of inulin-type hexasaccharide extracted from *Morinda officinalis* on PC12 cells against the lesion induced by corticosterone. *Life Sciences*.

[23] Olennikov D. N., Tankhaeva L. M., Rokhin A. V. (2011). Glucofructans from *Saussurea lappa* roots. *Chemistry of Natural Compounds*.

[24] Hyun S. K., Jung H. A., Min B. S., Jung J. H., Choi J. S. (2010). Isolation of phenolics, nucleosides, saccharides and an alkaloid from the root of *Aralia cordata*. *Natural Product Sciences*.

[25] Xu Z. J., Lin C. W., Liao M. F. (2011). Structure analysis of a new oligosaccharides BROS from *Blumea riparia*. *Chinese Journal of Organic Chemistry*.

[26] Xu Z. J., Lin C. W., Shi Y. (2011). Isolation and structure determination of a new oligosaccharide from *Blume riparia*. *Journal of Medicinal Plants Research*.

[27] Wang Y., Jiang R. Z., Li G. R. (2010). Structural and enhanced memory activity studies of extracts from *Panax ginseng* root. *Food Chemistry*.

[28] Wan D., Jiao L., Yang H., Liu S. (2012). Structural characterization and immunological activities of the water-soluble oligosaccharides isolated from the *Panax ginseng* roots. *Planta*.

[29] Zhou L. G., Yang C. Z., Li J. Q., Wang S. L., Wu J. Y. (2003). Heptasaccharide and octasaccharide isolated from *Paris polyphylla* var. *yunnanensis* and their plant growth-regulatory activity. *Plant Science*.

[30] Zhou L. G., Cao X. D., Zhang R. F., Peng Y. L., Zhao S. J., Wu J. Y. (2007). Stimulation of saponin production in *Panax ginseng* hairy roots by two oligosaccharides from *Paris polyphylla* var. *yunnanensis*. *Biotechnology Letters*.

[31] Vanhaecke M., van den Ende W., Lescrinier E., Dyubankova N. (2008). Isolation and characterization of a pentasaccharide from *Stellaria media*. *Journal of Natural Products*.

[32] Feng J., Zhao W. (2009). Complete ^1^Hand ^13^C NMR assignments of four new oligosaccharides and two new glycosides from *Periploca forrestii*. *Magnetic Resonance in Chemistry*.

[33] Feng J. Q., Zhang R. J., Zhou Y. (2008). Immunosuppressive pregnane glycosides from *Periploca sepium* and *Periploca forrestii*. *Phytochemistry*.

[34] Long X. H., Xu R., Zhang Y. H., Tan X. H., Sun Q. Y. (2012). A new oligosaccharide from *Periploca calophylla*. *Zhongguo Zhongyao Zazhi*.

[35] Wang L., Yin Z.-Q., Wang Y., Zhang X.-Q., Li Y.-L., Ye W.-C. (2010). Perisesaccharides A-E, new oligosaccharides from the root barks of *Periploca sepium*. *Planta Medica*.

[36] Lei T., Zhang L., Jiang H.-Y., Hu Y., Hong A.-H., Cen Y.-Z. (2011). A new pregnane glycoside and oligosaccharide from *Parabarium huaitingii*. *Journal of Asian Natural Products Research*.

[37] Li J., Jiang Y., Tu P. F. (2005). Tricornoses A-L, oligosaccharide multi-esters from the roots of *Polygala tricornis*. *Journal of Natural Products*.

[38] Tu H. H., Liu P., Mu L. (2008). Study on antidepressant components of sucrose ester from *Polygala tenuifolia*. *Zhongguo Zhongyao Zazhi*.

[39] She G. M., Ba Y. Y., Liu Y., Lv H., Wang W., Shi R. B. (2011). Absorbable phenylpropenoyl sucroses from *Polygala tenuifolia*. *Molecules*.

[40] Yong J., Tu P. F. (2004). Studies on the chemical constituents in root bark of *Polygala tenuifolia* (II). *China Journal of Chinese Materia Medica*.

[41] Ono M., Takamura C., Sugita F. (2007). Two new steroid glycosides and a new sesquiterpenoid glycoside from the underground parts of *Trillium kamtschaticum*. *Chemical and Pharmaceutical Bulletin*.

[42] Ling Y., Li Z., Chen M., Sun Z., Fan M., Huang C. (2013). Analysis and detection of the chemical constituents of Radix Polygalae and their metabolites in rats after oral administration by ultra high-performance liquid chromatography coupled with electrospray ionization quadrupole time-of-flight tandem mass spectrometry. *Journal of Pharmaceutical and Biomedical Analysis*.

[43] Fan P., Terrier L., Hay A.-E., Marston A., Hostettmann K. (2010). Antioxidant and enzyme inhibition activities and chemical profiles of *Polygonum sachalinensis* F.Schmidt ex Maxim (Polygonaceae). *Fitoterapia*.

[44] Zhang L. J., Liao C. C., Huang H. C., Shen Y. C., Yang L. M., Kuo Y. H. (2008). Antioxidant phenylpropanoid glycosides from *Smilax bracteat*a. *Phytochemistry*.

[45] Yan L. L., Gao W. Y., Zhang Y. J., Wang Y. (2008). A new phenylpropanoid glycosides from *Paris polyphylla* var. *yunnanensis*. *Fitoterapia*.

[46] Kuo Y. H., Hsu Y. W., Liaw C. C., Lee J. K., Huang H. C., Kuo L. M. Y. (2005). Cytotoxic phenylpropanoid glycosides from the stems of *Smilax china*. *Journal of Natural Products*.

[47] Wang W.-X., Li T.-X., Ma H., Zhang J.-F., Jia A.-Q. (2013). Tumoral cytotoxic and antioxidative phenylpropanoid glycosides in *Smilax riparia* A. DC. *Journal of Ethnopharmacology*.

[48] Nhiem N. X., van Kiem P., van Minh C. (2009). Phenylpropanoid glycosides from *Heterosmilax erythrantha* and their antioxidant activity. *Archives of Pharmacal Research*.

[49] Wang Y., Gao W. Y., Zhang T. J., Guo Y. Q. (2007). A novel phenylpropanoid glycosides and a new derivation of phenolic glycoside from *Paris Polyphylla* var. *yunnanensis*. *Chinese Chemical Letters*.

[50] Zhang J.-Y., Wang Y.-Z., Zhao Y.-L. (2011). Phytochemicals and bioactivities of *Paris* species. *Journal of Asian Natural Products Research*.

[51] Hashim N. H. N., Abas F., Shaari K., Lajis N. H. (2012). LC-DAD-ESIMS/MS characterization of antioxidant and anticholinesterase constituents present in the active fraction from *Persicaria hydropiper*. *LWT: Food Science and Technology*.

[52] Fan P. H., Hay A. E., Marston A., Lou H. X., Hostettmann K. (2009). Chemical variability of the invasive neophytes *Polygonum cuspidatum* Sieb. and Zucc. and *Polygonum sachalinensis* F. Schmidt ex Maxim. *Biochemical Systematics and Ecology*.

[53] Liu P., Hu Y., Guo D. H. (2010). Potential antidepressant properties of Radix Polygalae (Yuan Zhi). *Phytomedicine*.

[54] Wu J. F., Chen S. B., Chen S. L., Tu P. F. (2007). The chemical constituents of *Polygala hongkongensis* Hemsl. *Acta Pharmaceutica Sinica*.

[55] Song Y., Jiang Y., Bi D., Tian X., Liang L., Tu P. (2012). Chemical constituents from *n*-butanol extract of aerial part of *Polygala sibirica*. *Zhongguo Zhongyao Zazhi*.

[56] Ikeya Y., Takeda S., Tunakawa M. (2004). Cognitive improving and cerebral protective effects of acylated oligosaccharides in *Polygala tenuifolia*. *Biological and Pharmaceutical Bulletin*.

[57] Zheng C. J., Hu C. L., Ma X. Q., Peng C., Zhang H., Qin L. P. (2012). Cytotoxic phenylpropanoid glycosides from *Fagopyrum tataricum* (L.) Gaertn. *Food Chemistry*.

[58] Li X., Zhang Y. F., Yang L. (2012). Chemical profiling of constituents of *Smilacis glabrae* using ultra-high pressure liquid chromatography coupled with LTQ orbitrap mass spectrometry. *Natural Product Communications*.

[59] Sun T.-T., Zhang D.-W., Han Y., Dong F.-Y., Wang W. (2012). Smilasides M and N, two new phenylpropanoid glycosides from *Smilax riparia*. *Journal of Asian Natural Products Research*.

[60] Li J., Bi X., Zheng G., Hitoshi Y., Ikeda T., Nohara T. (2006). Steroidal glycosides and aromatic compounds from *Smilax riparia*. *Chemical & Pharmaceutical Bulletin*.

[61] Wang N., Yao X., Ishii R., Kitanaka S. (2003). Bioactive sucrose esters from *Bidens parviflora*. *Phytochemistry*.

[62] Nakamura S., Fujimoto K., Matsumoto T. (2013). Acylated sucroses and acylated quinic acids analogs from the flower buds of *Prunus mume* and their inhibitory effect on melanogenesis. *Phytochemistry*.

[63] Fujimoto K., Nakamura S., Matsumoto T. (2013). Medicinal flowers. XXXVIII. Structures of acylated sucroses and inhibitory effects of constituents on aldose reducatase from the flower buds of *Prunus mume*. *Chemical and Pharmaceutical Bulletin*.

[64] Nakamura S., Fujimoto K., Matsumoto T. (2013). Structures of acylated sucroses and an acylated flavonol glycoside and inhibitory effects of constituents on aldose reductase from the flower buds of *Prunus mume*. *Journal of Natural Medicines*.

[65] Dong L.-B., He J., Li X.-Y. (2011). Chemical constituents from the aerial parts of *Musella lasiocarpa*. *Natural Products and Bioprospecting*.

[66] Xiong Y., Deng K. Z., Guo Y. Q., Gao W. Y., Zhang T. J. (2009). New chemical constituents from the rhizomes of *Sparganium stoloniferum*. *Archives of Pharmacal Research*.

[67] Xiong Y., Deng K.-Z., Guo Y.-Q., Gao W.-Y., Zhang T.-J. (2008). Two new sucrose esters from *Sparganium stoloniferum*. *Journal of Asian Natural Products Research*.

[68] Chen B., Wang N. L., Huang J. H., Yao X. S. (2007). Iridoid and phenylpropanoid glycosides from *Scrophularia ningpoensis* Hemsl. *Asian Journal of Traditional Medicines*.

[69] Chen H., Zhou Y. Z., Qiao L. (2008). Two new compounds from *Cynanchum amplexicaule*. *Journal of Asian Natural Products Research*.

[70] Chang C.-L., Zhang L.-J., Chen R. Y. (2010). Antioxidant and anti-inflammatory phenylpropanoid derivatives from *Calamus quiquesetinervius*. *Journal of Natural Products*.

[71] Kawai Y., Kumagai H., Kurihara H., Yamazaki K., Sawano R., Inoue N. (2006). *β*-Glucosidase inhibitory activities of phenylpropanoid glycosides, vanicoside A and B from *Polygonum sachalinense* rhizome. *Fitoterapia*.

[72] Kiem P. V., Nhiem N. X., Cuong N. X. (2008). New phenylpropanoid esters of sucrose from *Polygonum hydropiper* and their antioxidant activity. *Archives of Pharmacal Research*.

[73] Wang K. J., Zhang Y. J., Yang C. R. (2005). Antioxidant phenolic constituents from* Fagopyrum dibotrys*. *Journal of Ethnopharmacology*.

[74] Cho N., Huh J., Yang H. (2012). Chemical constituents of *Polygala tenuifolia* roots and their inhibitory activity on lipopolysaccharide-induced nitric oxide production in BV2 microglia. *Journal of Enzyme Inhibition and Medicinal Chemistry*.

[75] Ren Q., Wu C. S., Zhang J. L. (2013). Use of on-line stop-flow heart-cutting two-dimensional high performance liquid chromatography for simultaneous determination of 12 major constituents in tartary buckwheat (*Fagopyrum tataricum* Gaertn). *Journal of Chromatography A*.

[76] Ren Q., Wu C. S., Ren Y., Zhang J. L. (2013). Characterization and identification of the chemical constituents from tartary buckwheat (*Fagopyrum tataricum* Gaertn) by high performance liquid chromatography/photodiode array detector/linear ion trap FTICR hybrid mass spectrometry. *Food Chemistry*.

[77] Li W., Sun Y. N., Yan X. T. (2013). NF-*κ*B inhibitory activity of sucrose fatty acid esters and related constituents from *Astragalus membranaceus*. *Journal of Agricultural and Food Chemistry*.

[78] Cheng J. T., He J., Li Y. (2012). Three new sucrose fatty acid esters from *Equisetum hiemale* L. *Helvetica Chimica Acta*.

[79] Jiang Y., Tu P.-F. (2003). Tenuifoliose Q, a new oligosaccharide ester from the root of *Polygala tenuifolia* Willd. *Journal of Asian Natural Products Research*.

[80] Yoshikawa M., Matsuda H., Morikawa T., Xie H. H., Nakamura S., Muraoka O. (2006). Phenylethanoid oligoglycosides and acylated oligosugars with vasorelaxant activity from *Cistanche tubulosa*. *Bioorganic & Medicinal Chemistry*.

[81] Tu P.-F., Shi H.-M., Song Z.-H., Jiang Y., Zhao Y.-Y. (2007). Chemical constituents of *Cistanche sinensis*. *Journal of Asian Natural Products Research*.

[82] Si C.-L., Lu Y.-Y., Qin P.-P., Sun R.-C., Ni Y.-H. (2011). Phenolic extractives with chemotaxonomic significance from the bark of *Paulownia tomentosa* var. *tomentosa*. *BioResources*.

[83] Wu J., Zhang S., Xiao Q. (2003). Phenylethanoid and aliphatic alcohol glycosides from *Acanthus ilicifolius*. *Phytochemistry*.

[84] Jiang Y., Tu P.-F. (2009). Analysis of chemical constituents in *Cistanche* species. *Journal of Chromatography A*.

[85] Otsuka H., Kuwabara H., Hoshiyama H. (2008). Identification of sucrose diesters of aryldihydronaphthalene-type lignans from *Trigonotis peduncularis*and the nature of their fluorescence. *Journal of Natural Products*.

[86] Suo M.-R., Yang J.-S., Liu Q.-H. (2006). Lignan oligosaccharide esters from *Eritrichium rupestre*. *Journal of Natural Products*.

[87] Tu P.-F., Song Z.-H., Shi H.-M., Jiang Y., Zhao Y.-Y. (2006). Arylethyl (=phenylethanoid) glycosides and oligosaccharide from the stem of *Cistanche tubulosa*. *Helvetica Chimica Acta*.

[88] Shyr M.-H., Tsai T.-H., Lin L.-C. (2006). Rossicasins A, B and rosicaside F, three new phenylpropanoid glycosides from *Boschniakia rossica*. *Chemical and Pharmaceutical Bulletin*.

[89] Fang R., Veitch N. C., Kite G. C., Howes M. J. R., Porter E. A., Simmonds M. S. J. (2011). Glycosylated constituents of *Iris fulva*and *Iris brevicaulis*. *Chemical & Pharmaceutical Bulletin*.

[90] Fu J., Zuo L., Yang J., Chen R., Zhang D. (2008). Oligosaccharide polyester and triterpenoid saponins from the roots of *Polygala japonica*. *Phytochemistry*.

[91] Cheng M.-C., Li C.-Y., Ko H.-C., Ko F.-N., Lin Y.-L., Wu T.-S. (2006). Antidepressant principles of the roots of *polygala tenuifolia*. *Journal of Natural Products*.

[92] Chang H. T., Tu P. F. (2007). New oligosaccharide esters and xanthone *C*-glucosides from *Polygala telephioides*. *Helvetica Chimica Acta*.

[93] Li J., Feng L., Dai J., Wang R., Nohara T. (2009). Chemical constituents from *Polygala telephioides*. *China Journal of Chinese Materia Medica*.

[94] Yin W.-P., Zhao T.-Z., Zhang H.-Y. (2008). A novel oligosaccharide ester from *Syringa pubescens*. *Journal of Asian Natural Products Research*.

[95] Valko M., Leibfritz D., Moncol J., Cronin M. T. D., Mazur M., Telser J. (2007). Free radicals and antioxidants in normal physiological functions and human disease. *The International Journal of Biochemistry & Cell Biology*.

[96] Krishnaiah D., Sarbatly R., Nithyanandam R. (2011). A review of the antioxidant potential of medicinal plant species. *Food and Bioproducts Processing*.

[97] Liu P., Hu Y., Guo D.-H. (2010). Antioxidant activity of oligosaccharide ester extracted from *Polygala tenuifolia* roots in senescence-accelerated mice. *Pharmaceutical Biology*.

[98] Hu Y., Liao H.-B., Dai-Hong G., Liu P., Wang Y.-Y., Rahman K. (2010). Antidepressant-like effects of 3,6′-disinapoyl sucrose on hippocampal neuronal plasticity and neurotrophic signal pathway in chronically mild stressed rats. *Neurochemistry International*.

[99] Hu Y., Liao H. B. O., Liu P., Guo D.-H., Rahman K. (2009). A bioactive compound from *Polygala tenuifolia* regulates efficiency of chronic stress on hypothalamic-pituitary-adrenal axis. *Pharmazie*.

[100] Jungbauer A., Medjakovic S. (2012). Anti-inflammatory properties of culinary herbs and spices that ameliorate the effects of metabolic syndrome. *Maturitas*.

[101] Xu Q., Wang Y., Guo S., Shen Z., Yang L. (2014). Anti-inflammatory and analgesic activity of aqueous extract of *Flos populi*. *Journal of Ethnopharmacology*.

[102] Kim K. S., Lee D. S., Bae G. S. (2013). The inhibition of JNK MAPK and NF-*κ*B signaling by tenuifoliside A isolated from *Polygala tenuifolia* in lipopolysaccharide-induced macrophages is associated with its anti-inflammatory effect. *European Journal of Pharmacology*.

[103] Lee J.-G., Yon J.-M., Lin C., Jung A. Y., Jung K. Y., Nam S.-Y. (2012). Combined treatment with capsaicin and resveratrol enhances neuroprotection against glutamate-induced toxicity in mouse cerebral cortical neurons. *Food and Chemical Toxicology*.

[104] Jin M. L., Park S. Y., Kim Y. H., Oh J.-I., Lee S. J., Park G. (2014). The neuroprotective effects of cordycepin inhibit glutamate-induced oxidative and ER stress-associated apoptosis in hippocampal HT22 cells. *NeuroToxicology*.

[105] Liu P., Hu Y., Li J. (2012). Protection of SH-SY5Y neuronal cells from glutamate-induced apoptosis by 3,6'-disinapoyl sucrose, a bioactive compound isolated from radix polygala. *Journal of Biomedicine and Biotechnology*.

[106] Li P.-B., Lin W.-L., Wang Y.-G., Peng W., Cai X.-Y., Su W.-W. (2012). Antidiabetic activities of oligosaccharides of *Ophiopogonis japonicus* in experimental type 2 diabetic rats. *International Journal of Biological Macromolecules*.

[107] Liu T., Yip Y. M., Song L. (2013). Inhibiting enzymatic starch digestion by the phenolic compound diboside A: a mechanistic and *in silico* study. *Food Research International*.

[108] Côté F., Hahn M. G. (1994). Oligosaccharins: structures and signal transduction. *Plant Molecular Biology*.

[109] Creelman R. A., Mullet J. E. (1997). Oligosaccharins, brassinolides, and jasmonates: nontraditional regulators of plant growth, development, and gene expression. *Plant Cell*.

[110] Jiao L., Wan D., Zhang X., Li B., Zhao H., Liu S. (2012). Characterization and immunostimulating effects on murine peritoneal macrophages of oligosaccharide isolated from *Panax ginseng C.A. Meyer*. *Journal of Ethnopharmacology*.

